# In Silico Approaches to Developing Novel Glycogen Synthase Kinase 3β (GSK-3β)

**DOI:** 10.3390/biomedicines11102784

**Published:** 2023-10-13

**Authors:** Shuchi Goyal, Manjinder Singh, Divya Thirumal, Pratibha Sharma, Somdutt Mujwar, Krishna Kumar Mishra, Thakur Gurjeet Singh, Ravinder Singh, Varinder Singh, Tanveer Singh, Sheikh F. Ahmad

**Affiliations:** 1 Chitkara College of Pharmacy, Chitkara University, Rajpura 140401, Punjab, India; shuchigoyal254@gmail.com (S.G.); somduttmujwar@gmail.com (S.M.); ravi.jaura@gmail.com (R.S.); 2 Chitkara University Institute of Engineering and Technology, Chitkara University, Rajpura 140401, Punjab, India; krishna.mishra@chitkara.edu.in; 3 Department of Pharmaceutical Sciences and Technology, Maharaja Ranjit Singh Punjab Technical University, Bathinda 151001, Punjab, India; varinderjassal17@gmail.com; 4 Department of Neuroscience and Experimental Therapeutics, College of Medicine, Texas A & M Health Science Center, Bryan, TX 77807, USA; tanveersingh1988@gmail.com; 5 Department of Pharmacology and Toxicology, College of Pharmacy, King Saud University, Riyadh 11451, Saudi Arabia

**Keywords:** GSK-3β, molecular dynamics, docking, scaffold, morphing, neurofibrillary tangles, Alzheimer’s disease, amyloid-beta

## Abstract

Alzheimer’s disease (AD) is caused by plaque agglomeration and entanglement in several areas of the neural cells, which leads to apoptosis. The main etiology of AD is senile dementia, which is linked to amyloid-beta (Aβ) deregulation and tau perivascular pathogeny. Hyperphosphorylated tau has a propensity for microtubules, which elevate the instability and tau-protein congregates, leading to accumulation of neurofibrillary tangles (NFTs). Tau hyperphosphorylation is susceptible to GSK-3, which has led to an emerging hypothesis regarding the pathogenesis of AD. Accordingly, attempts have been made to conduct investigations and achieve further advancements on new analogues capable of inhibiting the GSK-3 protein, which are currently in the clinical trials. In this analysis, we have evaluated certain GSK-3 inhibitor variants utilising scaffolding and framework devised techniques with pharmacological characteristics, accompanied by computational screenings (pharmacokinetics and docking). The structure-based designed analogues interacted effectively with the active amino acids of GSK-3β target protein. The in silico pharmacokinetic studies revealed their drug-like properties. The analogues with best interactions and binding scores will be considered in the future to completely demonstrate their potential relevance as viable GSK-3 inhibitors.

## 1. Introduction

In the United States, Alzheimer’s disease (AD) has become one of the most prevalent types of neurodegenerative dementia, and minority demographics are disproportionately affected. The phases of the disorder are characterised by abnormalities in the encoding and storage of recent memories. The subsequent phases are accompanied by further gradual alterations in intellect and conduct [1]. Around 50 million individuals nationwide were anticipated to have dementia in 2023, according to Alzheimer’s Disease International. This number is expected to quadruple by 2050, with two-thirds of those individuals residing in low-income and middle-income nations. Additionally, females are considerably more prone than males to succumb to Alzheimer’s disease, particularly after the age of 80 [2]. Because of an analogous amyloid β stress response, women are disproportionately inclined to be diagnosed with an enhanced tau strain [3,4]. Alzheimer’s disease (AD) along with various neural disorders, transmission abnormalities in the respiratory and circulation mechanisms that reduce the amount of oxygen reaching the brain, nutrition deficiencies, vitamin B12 deficiencies, malignancies, and other conditions all contribute to the deterioration of intellectual abilities [5]. The external amyloid plaques along with internal tau neurofibrillary tangles (NFTs) are considered to be the predominant histopathologic pathologies of AD, according to the research into the disease’s pathophysiology and neurological pathology that underlies the present investigation. The main component of the amyloid plaques is a highly insoluble and proteolysis-resistant polypeptide fibril created by the breakdown of β-amyloid (Aβ) [6]. To help the neural transit framework, the tau protein, which is a microtubule-associated protein, interacts with microtubules in the neural region. Axons in growth are stabilised by microtubules, which is essential for the pathology and mechanism of neurons. Unusually, hyperphosphorylated tau binds into intraneural tangles and creates intractable fibrils. This causes decoupling in the microtubules, which initiates the microtubule breakdown [7]. The detrimental and physiological alterations caused by the interactions of oligomers of the Aβ protein via glial cells and the neurons include mitochondrial dysfunction, pro-inflammatory cascade excitation, elevated tau phosphorylation along with oxidative tension, disruption of calcium metabolism, strengthened glycogen synthase kinase (GSK)-3 action, and activation of cell mortality and apoptosis in neurons [8].

Threonine, serine, and the amino acid residues next to proline are the three sites where the tau protein is phosphorylated. At certain residue sites, it binds to microtubules. The peptide is primarily hyperphosphorylated at positions T231, S235, and S265. Afterwards, the polypeptide forfeits its capacity to adhere to the microtubule and create neurofibrillary tangles [9].

For prevention, evaluation, and consequent therapy of Alzheimer’s disease patients, healthcare professionals must be able to quickly and accurately identify the manifestations and histology of the disorders that are linked to Alzheimer’s disease. This ability also permits the individuals who are caring for them to implement necessary behavioural modifications that may prolong the maintenance of their quality of life [10].

Aβ plaques and NFTs, which are key neuropathologic indicators of AD, are often found some time before neurological signs arise [11,12]. Such biomarkers indicating this underpinning pathophysiology have significant potential for the upfront screening of people who are most susceptible to acquiring MCI as a result of AD.

In the cognitive systems of AD individuals, hyperphosphorylated tau protein makes up the majority of NFTs, and the disease’s progression can be interpreted in terms of the NFTs’ morphological stages, which involve: (1) the pre-tangle period of a specific kind of NFT, where phosphorylated tau proteins gather in the somatodendritic space without the development of PHFs (paired helical filaments); (2) the phase of competent NFTs, which are distinguished by tau protein filament accumulation and the relocation of their nuclei to lateral regions of the soma, and (3) the extracellular tangles, also known as the “ghost NFT” phase, which develops after a synaptic lesion as a consequence of an abundance of filamentous tau protein that is partially proteolytically resistant [5]. The reason for the physiological alterations in Alzheimer’s disease (A, NFTs, and neuronal atrophy) is currently unclear. Numerous theories have been put forth about the primary factors in AD: a few contend that cholinergic dysfunction is a significant risk aspect for AD, whereas others contend that changes in the manufacturing and analysing of amyloid-protein are the primary triggering factors. However, there is currently no widely recognised hypothesis for the onset of AD [13,14].

**Figure 1:**
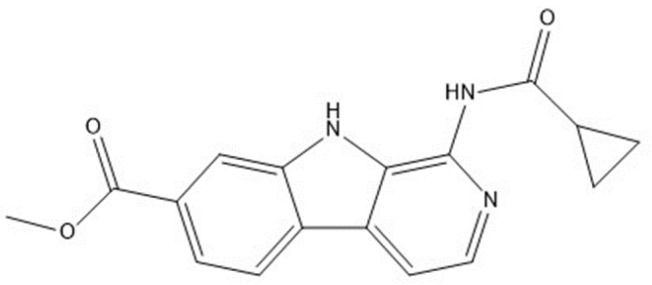
ZDWX-25 chemical structure.

Other important amino acid residues comprising the binding site of the GSK-3β enzyme are ASP133, Asp200, Cys199, Asn186, Glu97, Leu188, and Ala83. Among the varied crucial amino acids of the active site, the catalytic triad amino acids of GSK-3β could be the prime targets in the design of inhibitors [15,16]. In the current study, ZDWX-25 ((Methyl-1-(cyclopropanecarboxamido)-*9H*-pyrido[3,4-*b*]indole-7-carboxylate, Figure 1), a small GSK inhibitor used in clinical trials, was taken as lead molecule for further designing to reduce the GSK-3β levels in plasma and cerebrospinal fluid (CSF).

Through the use of scaffold-morphing methodologies, different fundamental equivalents of ZDWX-25 that had a high synthetic accessibility index were produced. The aforementioned analogues were subsequently tested for drug-likeness and BBB penetration using in silico pharmacokinetic methods. They were then subjected to molecular docking to examine their bonding affinity with the -secretase enzymes, which produced three intriguing prospects for the eventual generation of GSK-3 antagonist drugs.

## 2. Material and Methods

### 2.1. Clinical Trials Screening

Regarding the small-molecule GSK inhibiting agents, the clinical studies repository (clinicaltrials.gov.in) was searched. There were seven compounds discovered; these are given in Table 1. The majority of the compounds now being evaluated in clinics decrease the level of tau in CSF, but the studies have been halted because of toxicity to the liver and other serious unwanted reactions. Although ZDWX-25 has been demonstrated to be efficient at lowering tau concentrations in CSF along with plasma, resulting in a more gradual progression of cognitive decline and the absence of liver toxicity, the molecule’s adverse reactions included unusual dreams, allergic reactions, discomfort, diarrhoea, and infections of the upper respiratory tract [17,18].

### 2.2. Scaffold Morphing

Scaffold morphing, a medicinal science method for molecular framework changes, is currently utilised to create unique compounds with possibly superior features to maintain the fundamental efficacy. These drug-design methodologies are basically chemistry-driven and are restricted by the synthetic viability of novel scaffolds. These novel scaffolds have better features, such as water dissolution, an analogous logD, and higher residual plasma fractions, while maintaining powerful DprE1 inhibitory effects and potent antimycobacterial effectiveness. In contrast to the described azaindoles, an instance of benzimidazole molecule evaluated in a mouse TB model showed efficient execution. Numerous interacting affinities between benzimidazole and the azaindole scaffold are possible using in silico docking using DprE1. The development of these novel substrates for the utilisation of drug-sensitive as well as drug-resistant tuberculosis treatments is possible [19].

In scaffold morphing technique, the bio-isosteric replacement method was implemented in which bio-isosteres of various functional groups were substituted in a parent molecule to improve its potency along with pharmacokinetic profile [20]. In the present study, the bio-isosteric replacement in the parent molecule, ZDWX-25 was carried out using a web server MolOpt, a lately established web-tool for in silico drug designing [21]. This web server spontaneously creates numerous analogues based on bio-isosteric transformation rules with data mining, deep generative models, and similarity comparison. The overall bio-isosteric substitution approach, which includes replacing a molecule’s functional structures with its bio-isosteres, is utilised for scaffold morphing in order to increase the effectiveness and pharmacokinetics characteristic of one specific compound. Employing a publicly accessible online platform called MolOpt, the bio-isosteric alteration of arbidol was carried out during the present investigation. A newly created web resource for in silico designing drugs is MolOpt [20,21]. Equipped with bio-isosteric translation algorithms generated via data mining, deep generative theories, and similarity comparison, this online platform dynamically creates a number of analogs. Employing the open-source web server MolOpt, ZDWX-25 scaffold morphing was carried out in the present study. Implementing four built-in transformation rules—AI generative model, data mining, data mining (fast), and similarity comparison—this webserver produced 5347 compounds. Synthetic accessibility was used for arranging the derived ZDWX-25 analogs. Synthetic accessibility is graded on a scale of 1 (very easy) to 10 (very difficult) [22,23,24]. The generated analogues were screened using a threshold of 2.4, and the chosen compounds continued to undergo in silico pharmacokinetic tests.

### 2.3. In Silico Pharmacokinetic Predictions

Applying the online free digital swissADME application tool, which can be found at http://www.swissadme.ch, the pharmacokinetic characteristics of each of the chosen 100 compounds were assessed. Numerous molecular characteristics, notably physicochemical characteristics, lipophilicity, water solubility, pharmacokinetics, and drug likelihood characteristics, especially Lipinski’s rule of five (Ro5), were investigated [25]. BBB permeability was taken into account alongside relevant criteria when assessing the GSK inhibitory variants, because the drug that was being investigated was for Alzheimer’s disease. The analogs that showed BBB permeation were subsequently used for molecular docking investigations.

### 2.4. Molecular Docking Studies

Utilising the Biovia discovery studio program, molecular docking assays were conducted to investigate the affinities of the generated and categorised ZDWX-25 compounds with the GSK enzyme. The crystalline structures of the GSK protein was used for the molecular docking of the ZDWX-25 molecules, and an R-value of 0.281 was discovered by the RCSB-protein database library (PDB) (https://www.rcsb.org/) PDB ID: 4PTC [26,27]. The “macromolecule component” of the Biovia discovery studio application was used to synthesise the protein. The “small molecular component” of the discovery studio program was used to create the ligands. The dock ligands “CDOCKER” methodology was used to carry out the docking, while the molecular associations of the docked postures were recorded. For every ligand, a total of three interacted modalities could be created throughout the docking implementation procedure. The docking procedure was confirmed prior to docking the ligands by redocking a co-crystallised ligand bound to the GSK-3 protein. Equivalent procedures were used for the synthesis of the ligands and the docking analyses. It was possible to see how individual docked ligands interacted against the catalytic dyad (Val135 and Lys85) domains of the GSK-3 protein. We recorded the relationships, docking outcome, and bound energies.

### 2.5. Molecular Dynamic Simulations

By conducting a molecular dynamics (MD) simulation over an interval of 100 ns employing the molecular dynamics component of Schrodinger’s Desmond software 2023-2, the proposed analogues with favourable linking energy levels in comparison to the GSK 3β enzyme were additionally evaluated for their thermal equilibrium with reference to time [28,29,30,31]. By achieving the compound’s most desired configuration following interactions in relation to duration, the lowered or minimised energy simulations helped strengthen the protein–ligand interaction. Through examination of its simulated interaction, the linkage that persisted amongst the intricate ligand and the intended macromolecule for the entire 100 ns of simulations was examined [30,32]. This approach provided insight into key ligand interactions that can be used to determine a ligand’s affinities with greater precision. The component was initially constructed employing the orthorhombic box-shaped TIP3P solvable approach, and the input system’s ionic potential was changed using a 0.15 M salt solutions. The NPT consortium and a time interval of 1.0 fs were used to run the modelling procedure; the consistent temperature of 310 K and barometric pressure of 1.01325 bar were set through the Nose–Hoover Chain technique and the Martyn–Tobias–Klein method, respectively.

## 3. Results and Discussion

### 3.1. Scaffold Morphing through Bioisosteric Replacement

To create analogues with better pharmacokinetic, physicochemical, and pharmacologic features, the molecular framework of ZDWX-25 was designed using the molopt application sort feature. All four of the server’s available built-in bioisosteric substitution protocols were utilised. Table 2 lists the proportion of substitute locations and analogues obtained via each of the protocols. A total of 5347 compounds were produced. The produced analogues were arranged in order of increasing synthetic accessibility. Analogs with synthetic accessibility values of 2.4 were taken into consideration for additional research to maintain accurate analysis. The top 35 analogues on the basis of synthetic accessibility are given in Table 3. After arranging, 35 compounds were discovered to have a rating of 2.4. These 35 individual compounds were then sent for additional in silico ADME screening after the removal of analogs with comparable structures and attributes.

### 3.2. In Silico Pharmacokinetic Studies

This phase involved evaluating the pharmacokinetic and drug-likeness of 100 compounds based on their ADME characteristics. The configuration of the analogues to Lipinski’s rule of five ((molecular weight < 500; QPlogP_o/w_ < 5, H-B donors ≤ 5 and H-bond acceptors ≤ 10)) was used to evaluate the drug-like qualities of the compounds. Lipinski’s rule of five was found to be followed by all 100 compounds. The physicochemical and pharmacokinetic properties of these 100 substances were within permissible limits, and they also had high GI absorption. Among all evaluated analogues, 35 molecules were selected for further studies, as they had drug likeliness properties along with BBB permeability (Table 4) [4]. Indeed, the parent molecule (**ZDWX-25**) did not show brain permeability, despite the fact that the medicine is intended to treat Alzheimer’s disease and would function as a GSK-3β antagonist.

### 3.3. Molecular Docking

Analyses of the molecular docking interactions of the chosen analogues are provided in figures below. Most of the formed analogues out of the 36 compounds docked (35 analogues and **ZDWX-25**) interacted with the two catalytic dyad positions of the GSK-3 protein (Val135 and Lys85) via van der Waals interactions, pi-anion, halogen fluorine connections, or carbon–hydrogen bonding. Additionally, these compounds had strong associations with several critical GSK-3 amino acid residues, including Asp200, Asn186, Leu188, Glu97, Ala83, Cys199, Tyr134, and Glu137 (Table 5).

**Figure 2:**
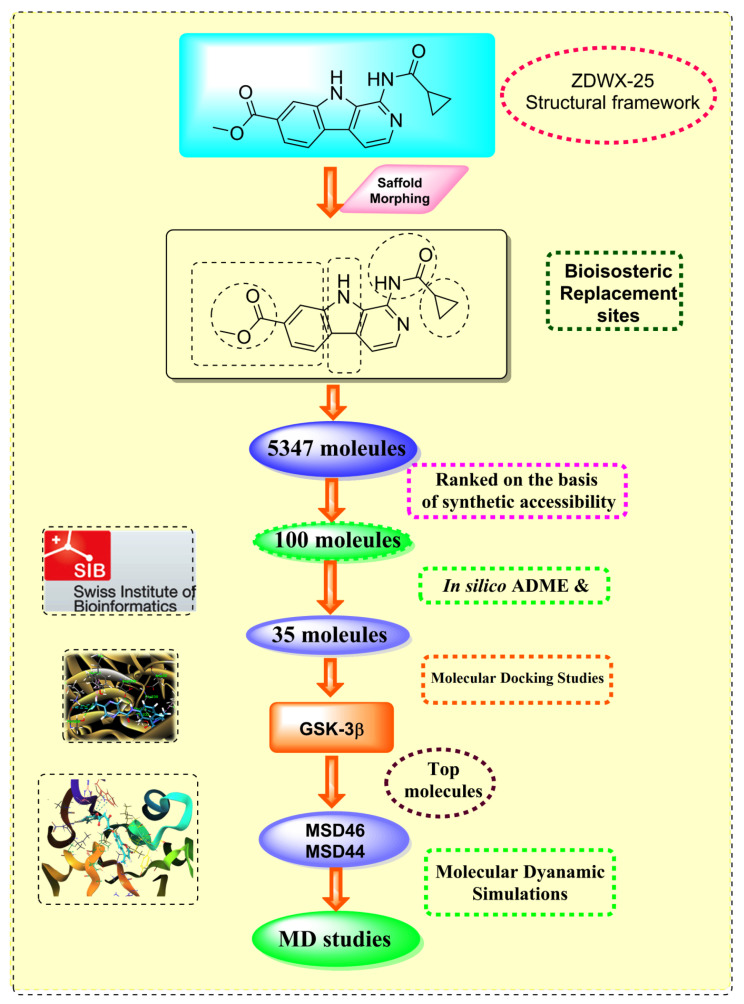
Generation of the potential glycogen synthase kinase 3β (GSK-3β) inhibitors via various steps involved in in silico study.

The leading three compounds with the highest docking values or lowest binding energies among all docked compounds were **MSD 46**, **MSD 44**, and **MSD 39** (Figure 2). The **MSD 46** bound the GSK-3 protein most effectively, with CDOCKER interaction energy of −40.8728, more than the parent molecule **ZDWX-25** (−36.4244).


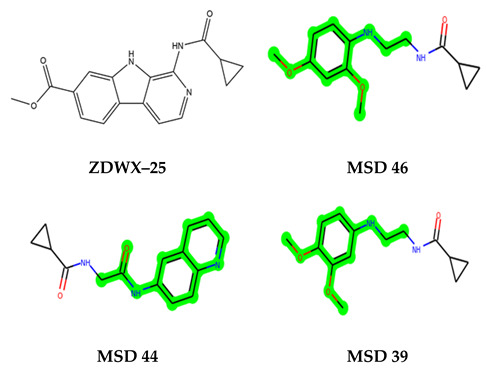


In lieu of the Methyl 1-(cyclopropanecarboxamido)-9*H*-pyrido[3,4-b]indole-7-carboxylate ring in **ZDWX-25**, the **MSD 46** component had an N-(2-((2,4-dimethoxyphenyl)amino)ethyl)cyclopropanecarboxamide ring. The remaining portion of the composition was the same. The **MSD 46** counterpart demonstrated conventional hydrogen contact with Lys85 and carbon–hydrogen bond interactions with the Val135 residues via the hydrogen of the phenyl ring. The phenyl ring interacted with Lys85 via conventional hydrogen bonding, with Ala83 via alkyl bonding, and with Val135, Asp200, Asp133, and Pro136 via carbon–hydrogen bonding. The methyl group interacted with VAL166 in an alkyl fashion. The interactions between the phenyl ring and the Leu188, Ile62, Tyr134, and Phe67 were amide–pi layered. The second-best docking score was achieved by the additional chosen analogue **MSD 44**, with CDOCKER interaction energy of −40.7119.

The methyl 1-(cyclopropanecarboxamido)-9*H*-pyrido[3,4-b]indole-7-carboxylate ring in **ZDWX-25** was replaced by an N-(2-Oxo-2-(quinolin-6-ylamino)ethyl)cyclopropanecarboxamide ring in the analogue. It displayed typical hydrogen bonding with Lys85 and Val135. The amide component of the carboxamide molecule interacted with Val135 through a typical hydrogen bond. The hydrogens of the cyclopropane ring interacted with Phe67 in a pi–pi stacking fashion. Cys199, Tyr134, and Val90 displayed pi–alkyl linkages with the methyl group. The compound interacted with all the nearby amino acids, including Asn186, Thr138, Glu137, Pro136, Leu188, and Ile62 through van der Waal interactions. The molecule interacted with Arg141, Asp133, Val110, and Leu132 via van der Waals interactions. The third-best docking score was achieved by the additional chosen analogue **MSD 39**, with CDOCKER interaction energy of −40.0165. In ZDWX-25, the methyl 1-(cyclopropanecarboxamido)-9*H*-pyrido[3,4-b]indole-7-carboxylate ring was replaced by an N-(2-((3,4-dimethoxyphenyl)amino)ethyl)cyclopropanecarboxamide ring in the analog. It displayed van der Waals interaction with Val135 as well as conventional hydrogen bonding and interactions with Lys85′s carbon–hydrogen bond. The carbonyl group of the carboxamide moiety interacted with Lys85 in a typical hydrogen bonding manner. The methyl group demonstrated alkyl connections with Ile62, Leu188, Tyr134, and Phe67, whereas the hydrogens of the pyridocarboxylate ring established carbon–hydrogen bonds with Val135 and Asp133 residues.

**Figure 3:**
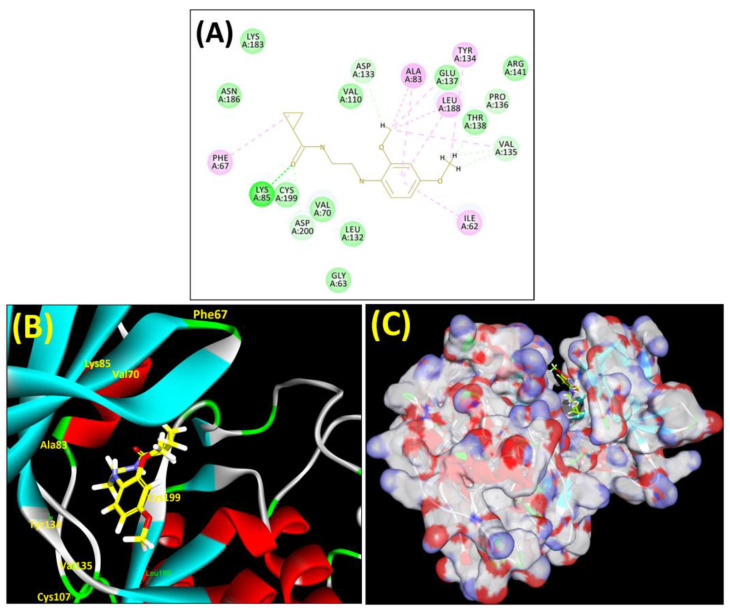
(**A**) A 2D representation of interactions of compound **MSD 46** with GSK-3β active site. (**B**) A 3D representation of different interactions of **MSD 46** with GSK-3β active site. (**C**) The surface view of GSK-3β enzyme and **MSD 46** in the active pocket.

**Figure 4:**
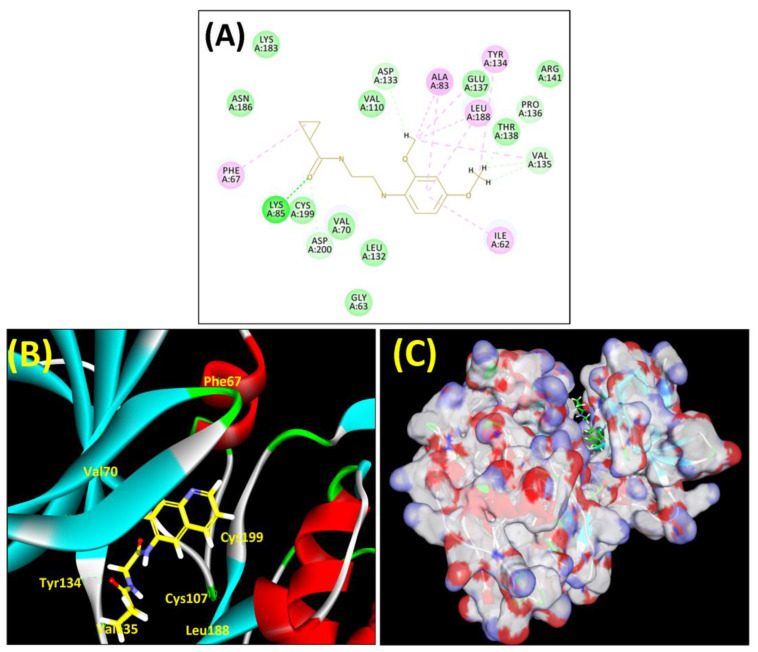
(**A**) A 2D representation of interactions of compound **MSD 44** with GSK-3β active site. (**B**) A 3D representation of different interactions of **MSD 44** with GSK-3β active site. (**C**) The surface view of GSK-3β enzyme and **MSD 44** in the active pocket.

**Figure 5:**
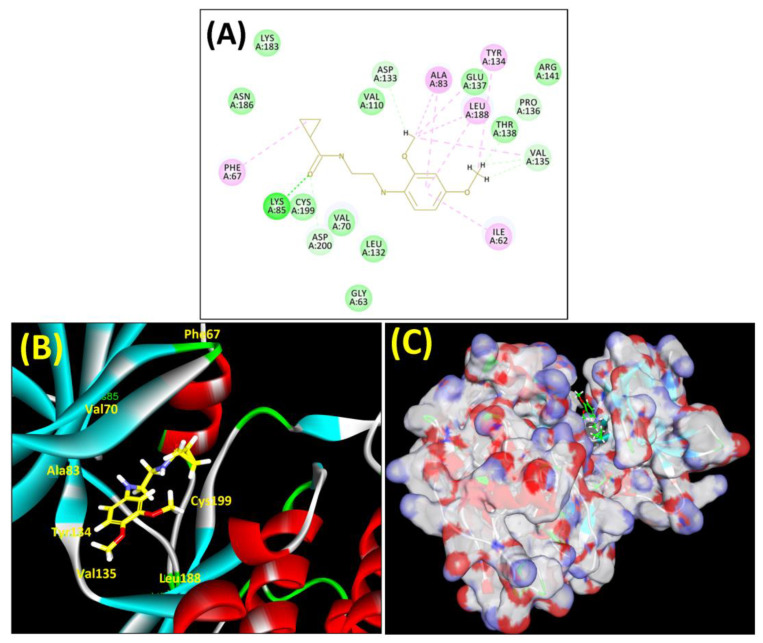
(**A**) A 2D representation of interactions of compound **MSD 39** with GSK-3β active site. (**B**) A 3D representation of different interactions of **MSD 39** with GSK-3β active site. (**C**) The surface view of GSK-3β enzyme and **MSD 39** in the active pocket.

All the neighbouring amino acids, including Val110, Lys183, Asn186, Cys199, Asp200, and Pro136, showed van der Waals interactions with the molecule. The **ZDWX-25** had CDOCKER interaction energy of −36.4244, which proved the newer analogues had better docking results, showing them to be promising candidates for further research in GSK-3β inhibition. The 2D, 3D, and surface views of **MSD 46**, **MSD 44,** and **MSD 39** are given in Figure 3A–C, Figure 4A–C and Figure 5A–C, respectively. The remaining amino acids in the area, including Val110, Lys183, Asn186, Cys199, Asp200, and Pro136, all exhibited van der Waals linkages with the molecule. The **ZDWX-25** demonstrated the novel analogues to be potential alternatives for more investigation in GSK-3 inhibition.

## 4. Molecular Dynamics Simulations

**Figure 6:**
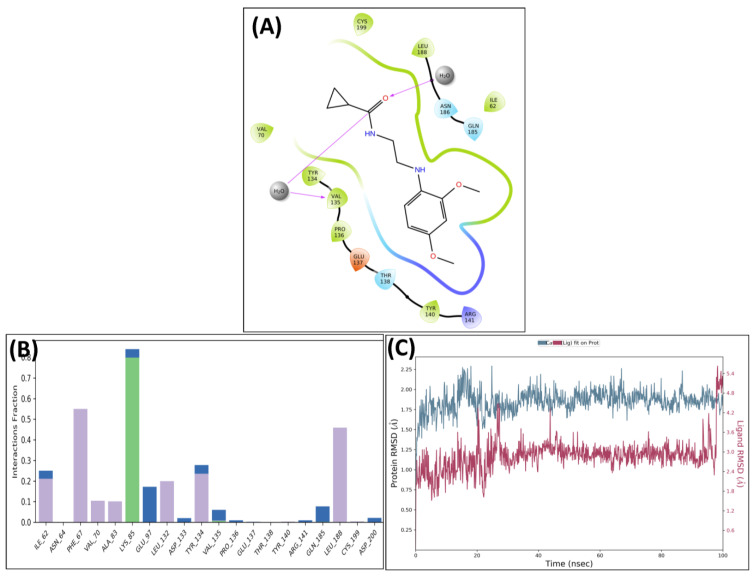
Post-MD H-bonds and hydrophobic interactions of **MSD 46** with GSK 3β. (**A**) Protein interaction fractions with the ligand (**MSD 46**) plot throughout the simulation. (**B**) Protein–ligand contacts plot of compound **MSD 46** with GSK 3β. (**C**) RMSD trajectory plot for compound **MSD 46**.

The leading two compounds were subjected to an MD simulation research in order to examine their thermodynamic stability, the dynamic conduct of the ligand–protein compound, and its impact on conformational modifications caused by ligand association with the activated domain of GSK 3 [33,34,35,36]. Though the significant correlations were preserved after MD, some new associations were also noticed. Analysis of the protein–ligand complex between GSK-3 and MSD 46 revealed interactions with **PHE67**, **VAL135**, **TYR134**, and **LEU188** via hydrophobic interactions. Additionally, using the **LYS85**, strong hydrogen bonding connections were seen (Figure 6A). The protein–ligand relation plot (Figure 6B), which represents these interactions, delivers an overview. Following MD simulations, the MSD 46 compound’s root mean square deviation (RMSD) with regard to the protein was determined and is depicted in Figure 6C.

**Figure 7:**
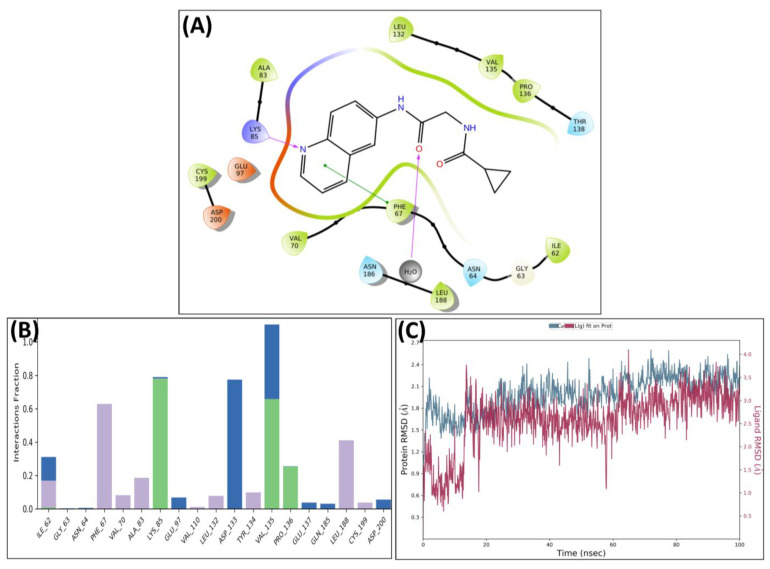
Post-MD H-bonds and hydrophobic interactions of **MSD 44** with GSK 3β. (**A**) Protein interaction fractions with the ligand (**MSD 44**) plot throughout the simulation. (**B**) Protein–ligand contacts plot of compound **MSD 44** with GSK 3β. (**C**) RMSD trajectory plot for compound **MSD 44**.

A similar pattern was observed in the complex of GSK 3β and **MSD 44**, which showed strong H-bonding interactions with **Lys85** and **Val135** along with hydrophobic interactions with **Phe67** and **Leu188**. The carbonyl of **MSD 44** also interacted with **Asp133** through water bridges (Figure 7A). The interactions are plotted in the protein–ligand contacts plot (Figure 7B). After MD simulation studies, the RMSD graph of MSD 44- GSK 3β complex was plotted against time (100 ns) (Figure 7C).

The RMSD plots of MSD 46 and MSD 44 revealed the stability of complexes with minor fluctuations (in the range of 1.2–2.0 Å) in the 100 ns time slot. The simulated stability indicated the inhibitory potential of the designed compounds **MSD 46** and **MSD 44** against GSK 3β. Of the two, the **MSD 46** was found to form a more stable complex, as it showed very little or no pulsation in the given time, whereas the **MSD 44** showed vibration during the first 20 ns within the binding cavity, and then stable conformation was achieved. Both analogues, **MSD 46** and **MSD 44**, showed a good degree of stability within the active site of the target protein during the simulation period, which was required to initiate the therapeutic response.

## 5. Conclusions

In silico structure-based drug designing along with scaffold morphing was effectively employed to identify the potent analogues of ZDWX-25 against GSK-3β. Initially, the bio-isosteric replacement was carried out on different suggested sites of ZDWX-25 in order to generate a library of its analogues. A small library of 5347 molecules was created, which was reduced to the 36 best molecules upon various screening steps. The leading molecules showed noteworthy docking behaviour in the binding site of GSK-3β. The most active molecules, **MSD 46**, **44**, and **39**, were identified with the highest docking scores and significant interactions against the target protein, GSK-3β. Further, **MSD 46** and **44** showed significant interactions with important amino acids of the active site post MD simulations. The pharmacokinetic parameters were also calculated for the designed molecules. The in silico ADME prediction confirmed the drug-like properties of these analogues. These novel designed molecules may be taken as lead potential GSK-3β inhibitors towards the treatment of Alzheimer’s disease.

## Figures and Tables

**Table 1:** Some of the small molecule inhibitors that are under clinical trials.

Molecule Name	Pharmacology and Clinical Consideration	Trial Status	Clinical Trial ID
GSK239512	Brain penetrant H3 receptor antagonist/inverse agonist	Phase II completed	NCT01009060
TRx0237	Tau stabilisers and aggregation inhibitors	Completed	NCT01689233
AADvac1	Tau stabilisers and aggregation inhibitors	Phase II-pending results	NCT02579252
Zagotenemab (LY3303560)	Capture and neutralise tau aggregate	Ongoing Phase II	NCT03518073
ANAVEX2-73	Anti-tau, anti-amyloid	Ongoing Phase II	NCT03790709
Solanezumab, Gantenerumab	Monoclonal antibodies	Recruiting clinical trials	NCT02008357, NCT01760005
Tideglusib (NP031112)	Inhibits GSK-3 irreversibly	Phase II	NCT01350362

**Table 2:** The corresponding number of analogs generated through bioisosteric replacement.

Protocol	No. of Replacements	Analogues Generated
AI generative model	09	1750
Data mining	06	728
Data mining (fast)	06	1150
Similarity comparison	09	1714

**Table 3:** Structures of top 35 designed analogues.

S.No	Compound ID	Structure	S.No	Compound ID	Structure
1.	MSD 1	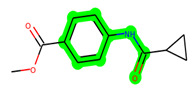	19.	MSD 23	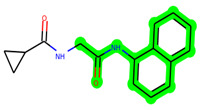
2.	MSD 3	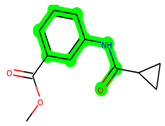	20.	MSD 24	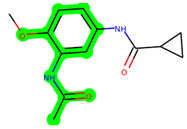
3.	MSD 4	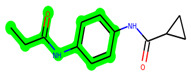	21.	MSD 25	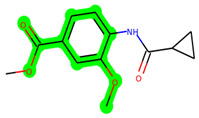
4.	MSD 5	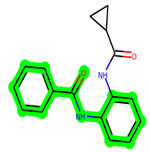	22.	MSD 27	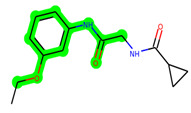
5.	MSD 6	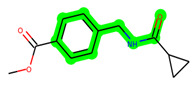	23.	MSD 30	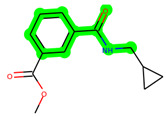
6.	MSD 7	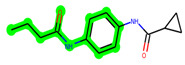	24.	MSD 31	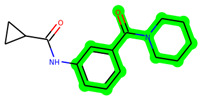
7.	MSD 8	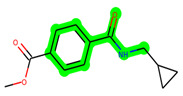	25.	MSD 33	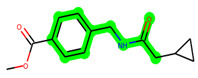
8.	MSD 9	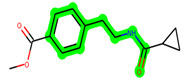	26.	MSD 35	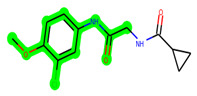
9.	MSD 10	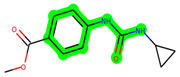	27.	MSD 38	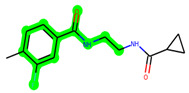
10.	MSD 11	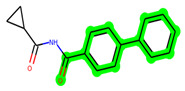	28.	MSD 39	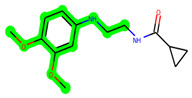
11.	MSD 12	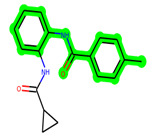	29.	MSD 42	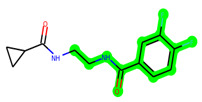
12.	MSD 13	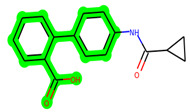	30.	MSD 43	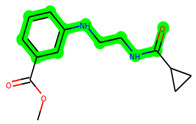
13.	MSD 15	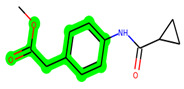	31.	MSD 44	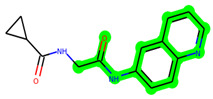
14.	MSD 16	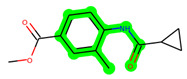	32.	MSD 46	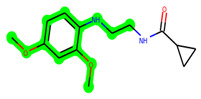
15.	MSD 17	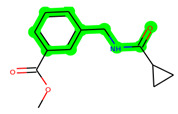	33.	MSD 47	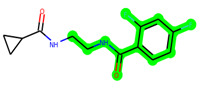
16.	MSD 18	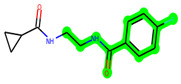	34.	MSD 49	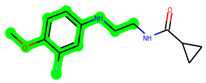
17.	MSD 19	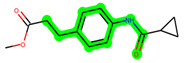	35.	MSD 50	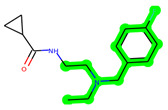
18.	MSD 20	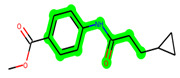			

**Table 4:** Predicted pharmacokinetic properties of best 35 designed analogues.

S.No.	CPD ID	MW	HB A	HBD	TPSA (Å)	Consensus Log P	Ali Log S	Lipinski Violations	Brain Permeability	GI Absorption
1.	MSD 1	219.24	3	1	55.4	1.89	−3.05	0	Yes	High
2.	MSD 3	219.24	3	1	55.4	1.83	−2.71	0	Yes	High
3.	MSD 4	232.28	2	2	58.2	1.7	−2.02	0	Yes	High
4.	MSD 5	280.32	2	2	58.2	2.58	−3.25	0	Yes	High
5.	MSD 6	233.26	3	1	55.4	1.77	−2.11	0	Yes	High
6.	MSD 7	246.3	2	2	58.2	2.02	−2.39	0	Yes	High
7.	MSD 8	233.26	3	1	55.4	1.99	−2.58	0	Yes	High
8.	MSD 9	247.29	3	1	55.4	2.11	−2.59	0	Yes	High
9.	MSD 10	234.25	3	2	67.43	1.67	−3.22	0	Yes	High
10.	MSD 11	265.31	2	1	46.17	2.92	−3.7	0	Yes	High
11.	MSD 12	294.35	2	2	58.2	2.92	−3.63	0	Yes	High
12.	MSD 13	281.31	3	2	66.4	2.66	−3.56	0	Yes	High
13.	MSD 15	233.26	3	1	55.4	1.82	−2.11	0	Yes	High
14.	MSD 16	233.26	3	1	55.4	2.09	−2.55	0	Yes	High
15.	MSD 17	233.26	3	1	55.4	1.92	−2.76	0	Yes	High
16.	MSD 18	250.27	3	2	58.2	1.66	−1.88	0	Yes	High
17.	MSD 19	247.29	3	1	55.4	2.09	−2.42	0	Yes	High
18.	MSD 20	247.29	3	1	55.4	2.58	−4.04	0	Yes	High
19.	MSD 23	268.31	2	2	58.2	2.04	−2.45	0	Yes	High
20.	MSD 24	248.28	3	2	67.43	1.47	−2.42	0	Yes	High
21.	MSD 25	249.26	4	1	64.63	1.87	−2.61	0	Yes	High
22.	MSD 27	262.3	3	2	67.43	1.57	−2.65	0	Yes	High
23.	MSD 30	233.26	3	1	55.4	2.08	−3.13	0	Yes	High
24.	MSD 31	272.34	2	1	49.41	2.23	−2.52	0	Yes	High
25.	MSD 33	247.29	3	1	55.4	2.11	−2.73	0	Yes	High
26.	MSD 35	282.72	3	2	67.43	1.77	−2.64	0	Yes	High
27.	MSD 38	264.3	3	2	58.2	1.97	−2.27	0	Yes	High
28.	MSD 39	264.32	3	2	59.59	1.66	−2.48	0	Yes	High
29.	MSD 42	268.26	4	2	58.2	1.93	−1.99	0	Yes	High
30.	MSD 43	262.3	3	2	67.43	1.67	−2.55	0	Yes	High
31.	MSD 44	269.3	3	2	71.09	1.49	−2.44	0	Yes	High
32.	MSD 46	264.32	3	2	59.59	1.72	−2.48	0	Yes	High
33.	MSD 47	268.26	4	2	58.2	1.9	−1.99	0	Yes	High
34.	MSD 49	252.28	3	2	50.36	2.01	−2.42	0	Yes	High
35.	MSD 50	264.34	3	1	32.34	2.54	−2.31	0	Yes	High
36.	ZDWX-25	309.33	4	2	84.08	2.14	−3.19	0	No	High

**Table 5:** Docking scores and key interactions of designed ZDWX-25 analogs with GSK-3β.

S.No.	Compound ID	Docking Score (kcal/mol)	Interactions
1.	[MSD46]	−40.8728	Val135, Lys85, Leu188, Ala83, Tyr134, Glu137, Arg141, Asp13, Asp 200,.
2.	[MSD44]	−40.7119	Val135, Lys85, Phe67, Leu132, Ala32, Val70, Cys199, Asp133, Tyr134, Ala83
3.	[MSD39]	−40.0165	Val135, Lys85, Tyr134, Ile62, Leu188, Asp200, Gln185, Pro136, Ala83
4.	[MSD23]	−39.4222	Lys85, Val135, Tyr134, Ala83, Leu188, Val70, Ile62, Phe67, Leu132
5.	[MSD31]	−38.6482	Val135, Lys85, Tyr134, Val70, Ala83, Leu188, Cys199, Thr138, Leu132
6.	[MSD33]	−38.5194	Val135, Lys85, Cys199, Ala83, Asp200, Phe67, Leu188, Val70, Ile62
7.	[MSD49]	−37.9704	Val135, Lys85, Ala83, Val110, Tyr134, Leu188, Pro136, Ile62, Asp200
8.	[MSD6]	−37.4285	Lys85, Val135, Cys199, Ala83, Leu188, Val70, Phe67, Ile62, Asp200
9.	[MSD35]	−37.1191	Lys85, Val135, Leu188, Ile62, Ala83, Leu132, Tyr134, Cys199, Val110, Asp200, Thr138
10.	[MSD9]	−36.8884	Lys85, Val135, Tyr134, Leu188, Phe67, Asp200, Cys199, Val70, Ala83
11.	ZDWX-25	−36.4244	Cys199, Pro136, Val135, Lys85, Leu188, Ala83, Tyr134, Glu137, Arg141, Asp13, Asp 200

## Data Availability

The data presented in this study are available within the article.
